# Supplementary material for: Resolving the full spectrum of human genome variation using Linked-Reads
Source: Genome Res. 2019 Apr;29(4):635–45. doi: 10.1101/gr.234443.118 (PMC6442396; doi:10.1101/gr.234443.118)
Supplement: Supplemental Material [file supp_gr.234443.118_Supplemental_longranger-2.2.zip › longranger-2.2.tar.bz2!/lr2.2-public/lib/stan/cmdstan/stan/doc/api/html/singular__values_8hpp_source.html]

Stan: src/stan/math/matrix/singular\_values.hpp Source File


|  |  |
| --- | --- |
|  | Stan  2.5.0  probability, sampling & optimization |


- Main Page
- Related Pages
- Namespaces
- Classes
- Files

- File List
- File Members

All Classes Namespaces Files Functions Variables Typedefs Enumerations Enumerator Friends Macros Pages

- src
- stan
- math
- matrix

singular\_values.hpp

Go to the documentation of this file.

1 #ifndef STAN\_\_MATH\_\_MATRIX\_\_SINGULAR\_VALUES\_HPP

2 #define STAN\_\_MATH\_\_MATRIX\_\_SINGULAR\_VALUES\_HPP

3

4 #include <stan/math/matrix/Eigen.hpp>

5

6 namespace stan {

7  namespace math {

8

17  template <typename T>

18  Eigen::Matrix<T,Eigen::Dynamic,1>

19  singular\_values(const Eigen::Matrix<T,Eigen::Dynamic,Eigen::Dynamic>& m) {

20  return Eigen::JacobiSVD<Eigen::Matrix<T,Eigen::Dynamic,Eigen::Dynamic> >(m)

21  .singularValues();

22  }

23

24  }

25 }

26 #endif

Eigen.hpp

stan::math::singular\_values

Eigen::Matrix< T, Eigen::Dynamic, 1 > singular\_values(const Eigen::Matrix< T, Eigen::Dynamic, Eigen::Dynamic > &m)

Return the vector of the singular values of the specified matrix in decreasing order of magnitude...

**Definition:** singular\_values.hpp:19


---

[ Stan Home Page ]

*© 2011–2014,
Stan Development Team.*
